# TMPSS: A Deep Learning-Based Predictor for Secondary Structure and Topology Structure Prediction of Alpha-Helical Transmembrane Proteins

**DOI:** 10.3389/fbioe.2020.629937

**Published:** 2021-01-25

**Authors:** Zhe Liu, Yingli Gong, Yihang Bao, Yuanzhao Guo, Han Wang, Guan Ning Lin

**Affiliations:** ^1^Shanghai Mental Health Center, Shanghai Jiao Tong University School of Medicine, School of Biomedical Engineering, Shanghai Jiao Tong University, Shanghai, China; ^2^Shanghai Key Laboratory of Psychotic Disorders, Shanghai, China; ^3^College of Intelligence and Computing, Tianjin University, Tianjin, China; ^4^School of Information Science and Technology, Institute of Computational Biology, Northeast Normal University, Changchun, China

**Keywords:** protein secondary structure, protein topology structure, deep learning, alpha-helical transmembrane proteins, long short-term memory networks

## Abstract

Alpha transmembrane proteins (αTMPs) profoundly affect many critical biological processes and are major drug targets due to their pivotal protein functions. At present, even though the non-transmembrane secondary structures are highly relevant to the biological functions of αTMPs along with their transmembrane structures, they have not been unified to be studied yet. In this study, we present a novel computational method, TMPSS, to predict the secondary structures in non-transmembrane parts and the topology structures in transmembrane parts of αTMPs. TMPSS applied a Convolutional Neural Network (CNN), combined with an attention-enhanced Bidirectional Long Short-Term Memory (BiLSTM) network, to extract the local contexts and long-distance interdependencies from primary sequences. In addition, a multi-task learning strategy was used to predict the secondary structures and the transmembrane helixes. TMPSS was thoroughly trained and tested against a non-redundant independent dataset, where the Q3 secondary structure prediction accuracy achieved 78% in the non-transmembrane region, and the accuracy of the transmembrane region prediction achieved 90%. In sum, our method showcased a unified model for predicting the secondary structure and topology structure of αTMPs by only utilizing features generated from primary sequences and provided a steady and fast prediction, which promisingly improves the structural studies on αTMPs.

## Introduction

Membrane proteins (MPs) are pivotal players in several physiological events, such as signal transduction, neurotransmitter adhesion, ion transport, etc. (Goddard et al., 2015; Roy, 2015). While transmembrane proteins (TMPs), as an essential type of MPs, span the entire biological membrane with segments exposed to both the inside and the outside of the lipid bilayers (Stillwell, 2016). As the major class of TMPs, alpha-helical TMPs are given great pharmacological importance, accounting for about 60% of known drug targets in the current benchmark (Wang et al., 2019). Nevertheless, the difficulties of acquiring their crystal structures always stand in our way due to their low solubilities in the buffers typically used in 2D-PAGE (Butterfield and Boyd-Kimball, 2004; Nugent et al., 2011). All of this is calling for accurate computational predictors.

Predicting alpha-helical TMPs' tertiary structure directly from amino acid sequences has been a challengeable task in computational biology for many years (Yaseen and Li, 2014), but some indirect measures may be worth considering. Since Pauling et al. (1951) performed the first protein secondary structure prediction in 1951, many indicators on the secondary structure level of proteins, such as topology structure (Wang et al., 2019), surface accessibility (Lu et al., 2019), have been demonstrated to be strongly associated with the 3D information of TMPs. Specifically, the secondary structure helps to identify function domains and guides the design of site-specific mutation experiments (Drozdetskiy et al., 2015), whereas the topology structure can help reveal the relative position relationship between TMPs and membranes (Tusnady and Simon, 2001). Generally, the performance of protein secondary structure prediction can be measured by Q3 accuracy in a 3-class classification, i.e., helix (H), strand (E), and coil (C), or Q8 accuracy in an 8-class classification under a more sophisticated evaluation system. Q3 is preferred according to its low cost and close ability in depicting the secondary structure compared with Q8.

Progress in the structure prediction for MPs is slower than that for soluble proteins (Xiao and Shen, 2015). At present, state-of-the-art methods aiming at predicting the secondary structure based on primary sequences, such as SSpro/ACCpro 5 (Magnan and Baldi, 2014), JPred4 (Drozdetskiy et al., 2015), PSIPRED 4 (Buchan and Jones, 2019), and MUFOLD-SSW (Fang et al., 2020), are all trained on soluble protein-specific datasets. However, none of those mentioned methods can simultaneously predict the secondary structure and topology structure of alpha-helical TMPs. More specifically, existing tools could not distinguish transmembrane helices of TMPs from non-transmembrane ones and, in-term, would weaken the TMPs' structure prediction specificity. Another common challenge among the available methods is that features fed into these models are often too miscellaneous, making the model prediction low efficient and even difficult for users to understand. Thus, a more suitable and practical tool for assisting the structure prediction of TMPs is greatly needed.

**Figure 1:**
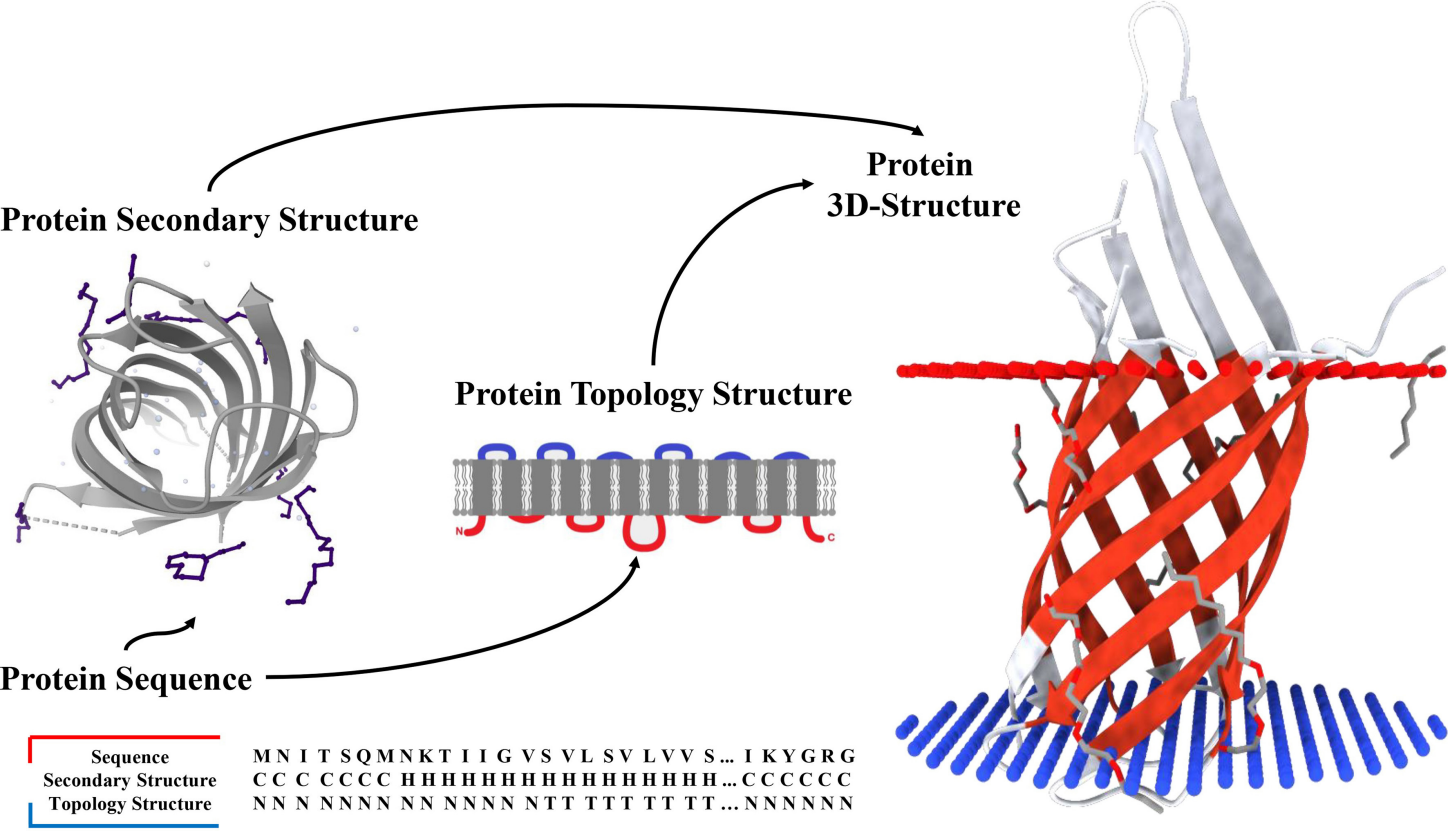
Secondary structure and topology structure prediction of alpha-helical transmembrane proteins.

Deep learning has been employed in several protein sequence classification problems (Lv et al., 2019; Wei et al., 2019; Zeng et al., 2020). Here, we proposed a deep learning-based predictor named TMPSS to predict the secondary structure and topology structure of alpha-helical TMPs simultaneously using amino acid sequences. Equipped with a robust network and carefully screened input features, TMPSS ignored input length restriction and achieved the highest output efficiency compared with other state-of-the-art methods with an acceptable Q3 performance of secondary structure prediction in the full chain (see Figure 1). In addition, our TMPSS achieved the Q3 of a whopping 0.97 in the transmembrane region, suggesting that almost all the transmembrane helices were identified. Moreover, TMPSS also significantly outperformed other existing topology structure predictors with the prediction accuracy of 0.90 and the Matthew Correlation Coefficient (MCC) of 0.76 using an independently generated dataset. TMPSS implemented a deep neural network by grouped multiscale Convolutional Neural Networks (CNNs) and stacked attention-enhanced Bidirectional Long Short-Term Memory (BiLSTM) layers for capturing local contexts and global dependencies, respectively. We also utilized the multi-task learning technique to improve prediction performance by considering the mutual effects between different protein properties. We have released TMPSS as a publicly available prediction tool for the community. The pre-trained model and support materials are both available at https://github.com/NENUBioCompute/TMP-SS.

## Materials and Methods

### Benchmark Datasets

As illustrated above, none of the existing secondary structure predictors available today are specific to TMPs. Thus, it is necessary to create unique datasets that contain only alpha-helical TMPs for targeted research. The Protein Data Bank of transmembrane proteins (PDBTM) (Kozma et al., 2012), the first up-to-date and comprehensive TMP selection of the Protein Data Bank (PDB) (Burley et al., 2017), was chosen to construct our datasets. We downloaded 4,336 alpha-helical TMPs from PDBTM (version: 2020-2-7) and removed the chains that contained unknown residues (such as “X”) and whose length was <30 residues.

To reduce the redundancy of data and avoid the influence of homology bias (Zou et al., 2020), we utilized CD-HIT (Fu et al., 2012) with a 30% sequence identity cut-off and obtained 911 protein chains. These protein chains were then randomly divided into a training set of 811 chains, a validation set of 50 chains, and a test set (named “TEST50”) of 50 chains. Secondary structure labels were obtained by the DSSP program (Kabsch and Sander, 1983) through PDB files, and topology structures were collected from PDBTM. All the experiments were conducted on five-fold cross-validation to gauge its generalization performances (Walsh et al., 2016). The results were used to evaluate our model and compare against other predictors. The overview of AA composition of the training set, validation set, and TEST50 is shown in Table 1.

**Table 1:** Overview of AA composition of the training set, validation set, and TEST50.

**3-State**	**8-State**	**Training set**		**Validation set**		**TEST50**	
Helices	G	60.1%	5,090	59.0%	438	55.1%	317
	H		119,987		7,931		7,897
	I		3,101		254		192
Strands	E	6.3%	12,226	6.5%	853	8.9%	1,240
	B		1,295		103		110
Coils	C	33.5%	34,372	34.5%	2,298	36.0%	2,607
	S		17,861		1,332		1,397
	T		19,195		1,409		1,488

### Features and Input Encoding

Features are the key issue for the machine learning tasks (Patil and Chouhan, 2019; Zhang and Liu, 2019). Prediction of alpha-helical TMPs' secondary structure and topology structure at the residue level is formulated as follows: for a given primary protein sequence of an alpha-helical TMP, a sliding window whose length is *L* residues is used to predict the secondary structure and topology structure of the central residue. For example, if *L* is 19, each protein will be sliced into fragments of 19 amino acids. Providing valuable input features to deep learning networks is of great importance to make predictions more accurate. Here, we carefully selected two encoding features to represent the protein fragment: one-hot code and HHblits profile (Remmert et al., 2012).

The first set came from the protein profiles generated by HHblits, which is faster, almost twice as sensitive, and provides more accurate evolutionary information for protein sequence than PSI-BLAST (Steinegger et al., 2019). We found the best results against the database named uniprot20_2016_02 with three iterations, an E-value threshold of 0.001, and other default settings. The obtained *H*_*hhm*_ matrix consisted of 31 dimensions, 30 of which were HMM profile values and one reflected *NoSeq* label (representing a gap) (Fang et al., 2018) at the last column. Each of *H*_*ij*_ in the matrix was scaled by a variation of sigmoid function [see Equation (1)], making the distribution of features more uniform and reasonable.

(1) $$\begin{array}{r} f\left(\text{t}\right)=\;\frac{10}{1+e^{-\frac{t}{2000}}}\; \end{array}$$

We then adopted a 21-dimensional matrix *O*_*onehot*_ as our second set containing a simple one-hot encoding of 20 positions with one *NoSeq* label. The past research suggested that one-hot encoding was straightforward to generate and has been successfully used in protein structure prediction-associated tasks (Ding and Li, 2015). Therefore, we used 19 dimensional “0” vector with a “1” to represent AA at the index of a particular protein sequence. We mapped each protein fragment sliced by the sliding window with this encoding strategy into an undisturbed coding within local position information.

### Model Design

#### Network Architecture

**Figure 2:**
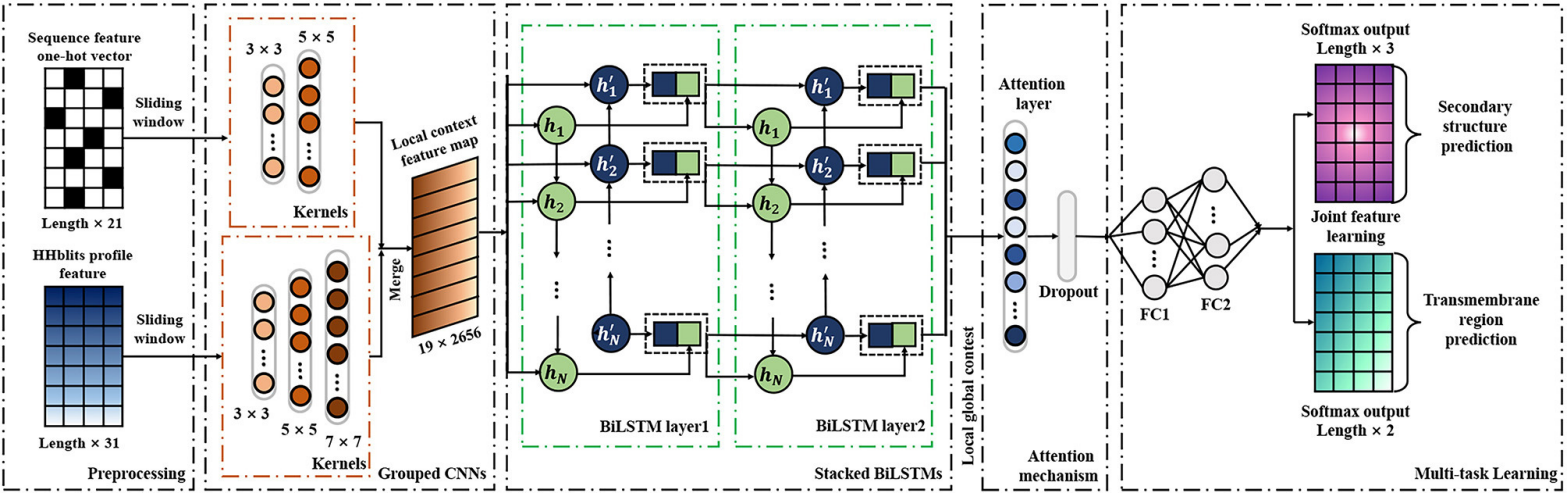
Diagram of TMPSS.

As a deep learning-based predictor, TMPSS can predict the secondary structure and topology structure of alpha-helical TMPs simultaneously. As we can see in Figure 2, the four parts of our model are feature-integration layers for input feature preprocessing, grouped multiscale CNN layers, attention-enhanced BiLSTM layer, and fully-connected layers by two softmax outputs in the end.

Our network's input carried two types of features generated from primary sequences, amino acid features, and profile features. These preprocessed features were fed into a grouped multiscale CNN layer to capture local position information and prevent their mutual interferences at the same time. Then, the merged CNN output flew into two stacked BiLSTM layers, which turned out to be skilled in extracting long-term dependencies and global information (Zhou et al., 2016). We also proposed the attention mechanism as a simple dense layer to help LSTM know which unit's output should be paid more attention. At the end of the components mentioned above, there were two fully-connected hidden layers with a softmax-activated output layer, which performed a 3-category secondary structure and 2-category topology structure classification. More details of grouped multiscale CNNs and attention-enhanced BiLSTM are discussed in the Supplementary Material.

#### Implementation Details

Our model was implemented, trained, and tested using the open-source software library Keras (Gulli and Pal, 2017) and Tensorflow (Abadi et al., 2016) on an Nvidia 1080Ti GPU. Main hyperparameters, such as sliding window length, training dropout rate, and number of LSTM units, were explored, and an early stopping strategy and a save-best strategy were adopted (Fang et al., 2018). When the validation loss did not reduce in 10 epochs during training time, the training process would be stopped, and the best model parameters would be saved. In all cases, the weights were initialized by default setting in Keras; the parameters were trained using an Adam optimizer (Bello et al., 2017) to change the learning rate during model training dynamically. Furthermore, batch normalization layers (Ioffe and Szegedy, 2015) and a Dropout layer (Gal et al., 2017) (rate = 0.30) were utilized since they were both skilled in avoiding the network from overfitting and improving the speed of the training process effectively. We set the sliding window's length as 19 residues and put 700 units in each LSTM layer according to the hyperparameter tuning results in this study.

### Performance Evaluation

A commonly used evaluation metric for both secondary structure and topology structure prediction based on the residue level is accuracy (ACC), and in particular, Q3 was widely used as a performance metric for 3-category secondary structure prediction (Fang et al., 2017). To quantitatively evaluate the performance of TMPSS and other predictors at the residue level, they were assessed by six measures, including accuracy, recall, precision, specificity, MCC, and F-measure (Tan et al., 2019; Yang et al., 2019; Zhu et al., 2019). The calculation formulas of these evaluation parameters were illustrated as follows:

(2) $$\begin{array}{r} Accuracy=\;\frac{TN+TP}{TP+FN+FP+TN} \end{array}$$

(3) $$\begin{array}{r} Recall=\frac{TP}{TP+FN}\; \end{array}$$

(4) $$\begin{array}{r} Precision=\frac{TP}{TP+FP}\; \end{array}$$

(5) $$\begin{array}{r} Speci\mathrm{fi}city=\frac{TN}{FP+TN} \end{array}$$

(6) $$\begin{array}{r} MCC=\frac{TP\times TN-FP\times FN}{\sqrt{\left(TP+FP\right)\left(TP+FN\right)\left(TN+FP\right)\left(TN+FN\right)}}\; \end{array}$$

(7) $$\begin{array}{r} F-measure=2\times \frac{Recall\times Precision}{Recall+Precision}\; \end{array}$$

where TN, TP, FN, and FP, respectively denoted true negative, true positive, false negative, and false positive samples.

## Results

### Prediction Performance Analysis at the Residue Level

**Figure 3:**
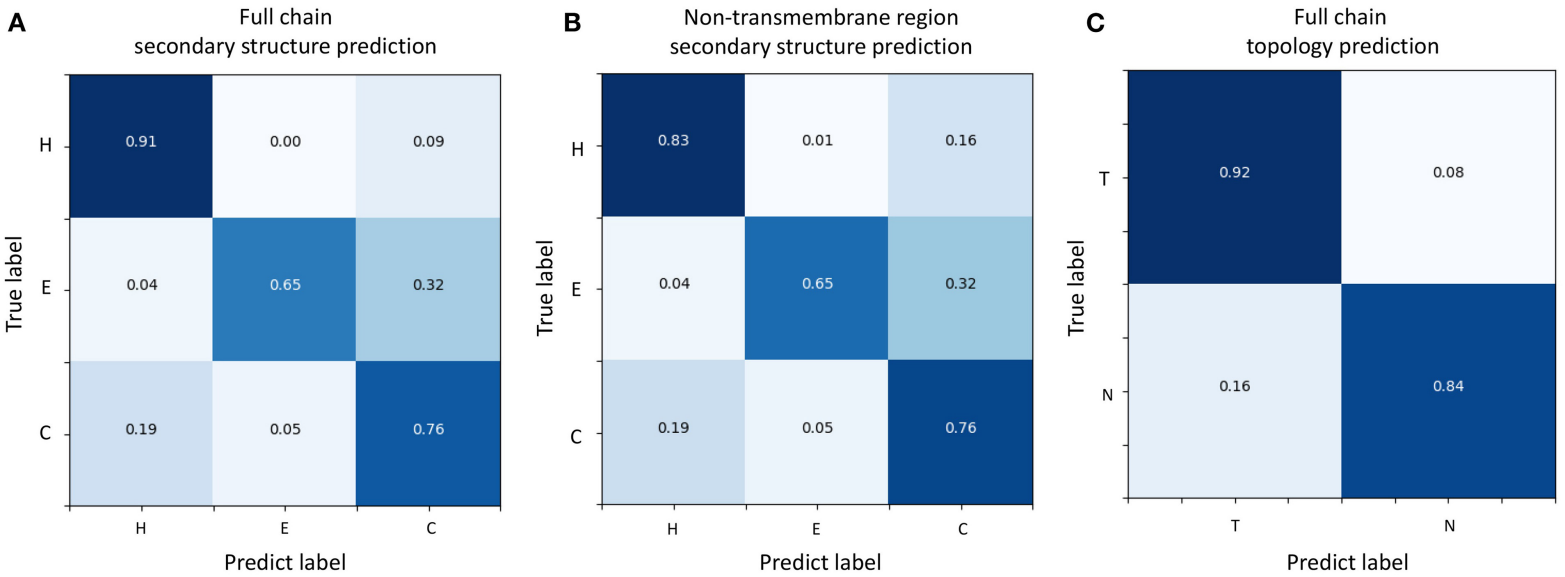
Confusion matrices of TMPSS's prediction performance. **(A)** Confusion matrix of secondary structure prediction in the full chain. **(B)** Confusion matrix of secondary structure prediction in the non-transmembrane region. **(C)** Confusion matrix of topology structure prediction in the full chain.

**Figure 4:**
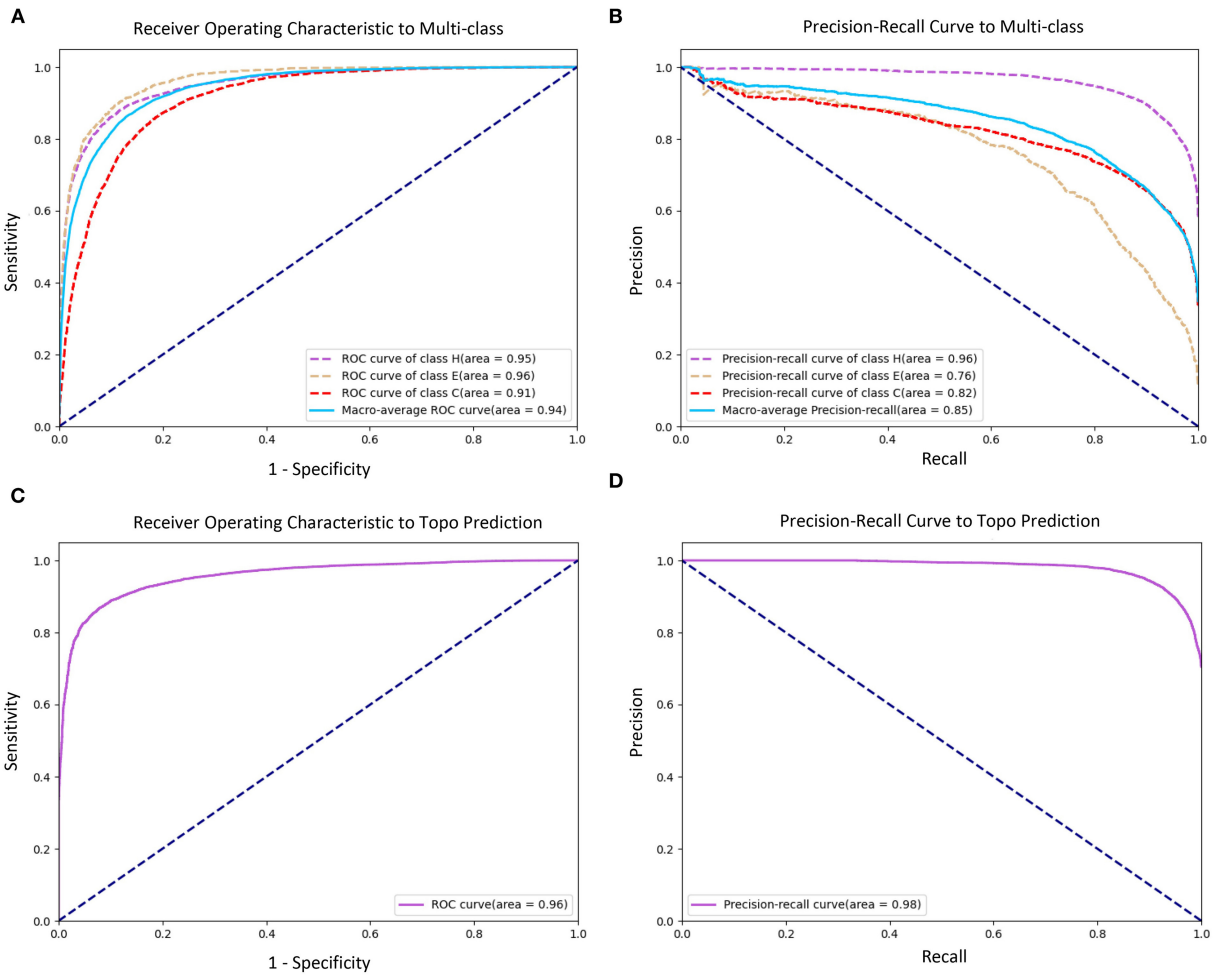
Receiver Operating Characteristic (ROC) and Precision–Recall (PR) curves of prediction performance. **(A)** ROC curve of secondary structure prediction. **(B)** PR curve of secondary structure prediction. **(C)** ROC curve of topology structure prediction. **(D)** PR curve of topology structure prediction.

To evaluate the prediction performance of each category in both two classification tasks at the residue level, we used the confusion matrices (see Figure 3), Receiver Operating Characteristic (ROC) curves, and Precision–Recall (PR) curves (see Figure 4) to visualize the predict results of TMPSS on TEST50. As illustrated in Table 1, TEST50 contains a total of 15,248 residues labeled by “H” (helix), “E” (strand), or “C” (coil) in secondary structure prediction and “T” (transmembrane helix) or “N” (non-transmembrane residue) in topology structure prediction.

Figures 3A,B shows the confusion matrices of secondary structure prediction in the full chain and non-transmembrane region, respectively. As we can see, class “H” was predicted with great precision in different regions of TMPs, but the results of class “E” and class “C” were less satisfactory. A similar experimental phenomenon existed in Figures 4A,B simultaneously. Helices account for the largest proportion and make the prediction more significant by considering our dataset's characteristics. The matrices demonstrate that TMPSS did well in both full chain and non-transmembrane region prediction of secondary structure on TEST50, confirming it to be a suitable secondary structure predictor for TMPs.

As for topology structure prediction, TMPSS is also an effective method. The confusion matrix of topology structure prediction in the full chain (see Figure 3C) proves that the output results performed well, whether for class “T” or class “N.” The ROC and PR curves (see Figures 4C,D) also support the above conclusion. After doing a thorough analysis of TMPSS's prediction performance at the residue level on TEST50, it can be seen that TMPSS is a reliable and convenient tool for predicting the secondary structure and topology structure of alpha-helical TMPs synchronously.

### Assessment of Multiple Predictors on TEST50

We tested TMPSS against SSpro5 (Magnan and Baldi, 2014) (with templates), PSIPRED 4 (Buchan and Jones, 2019), RaptorX-Property (Wang et al., 2016a), Porter 5 (Torrisi et al., 2019), DeepCNF (Wang et al., 2016b), Spider3 (Heffernan et al., 2017), SPOT-1D (Hanson et al., 2019), MUFOLD-SSW (Fang et al., 2020), and JPred4 (Drozdetskiy et al., 2015) on the TEST50 we created (see Table 2). Experimental results illustrated that SSpro5 (with templates) was the most accurate 3-state predictor in our tests on TEST50 in the full chain with a Q3 of 0.90. It might be probably because of the contribution of templates. However, apart from SSpro5 (with templates), the remaining servers performed similarly with the maximum Q3 deviation of 0.02, and some servers, such as JPred4, even performed worse. Many methods refused to accept sequences of more than a certain length. By comparison, TMPSS was user-friendly with no length limitation of input and had the highest output efficiency among the existing methods with an acceptable Q3 of 0.84 in the full chain.

**Table 2:** Comparison of TMPSS with previous secondary structure predictors on TEST50 in the full chain.

**Method**	**Class**	**R**	**P**	**S**	**MCC**	**F**	**Full chain SS Q3**	**Limitation of input length (residues)**	**Time cost (min)**
SSpro5 (with templates)	H	0.908	**0.942**	**0.923**	**0.826**	**0.925**	**0.90**	Limited to 1,500	980
	E	**0.908**	**0.778**	0.975	**0.824**	0.838			
	C	**0.870**	**0.854**	**0.926**	**0.792**	**0.862**			
PSIPRED 4	H	0.907	0.880	0.829	0.741	0.893	0.83	Limited to 1,500	490
	E	0.726	0.735	0.975	0.705	0.731			
	C	0.731	0.770	0.891	0.631	0.750			
RaptorX-Property	H	0.897	0.910	0.877	0.772	0.903	0.85	–	114
	E	0.771	0.761	0.977	0.743	0.766			
	C	0.786	0.770	0.883	0.666	0.778			
Porter 5	H	0.919	0.893	0.849	0.773	0.906	0.85	–	1,035
	E	0.757	0.763	0.977	0.737	0.760			
	C	0.758	0.796	0.903	0.670	0.777			
DeepCNF	H	0.867	0.908	0.879	0.741	0.887	0.83	–	3,000
	E	0.741	0.703	0.970	0.694	0.722			
	C	0.791	0.743	0.864	0.645	0.766			
Spider3	H	0.927	0.883	0.831	0.766	0.904	0.85	–	720
	E	0.751	0.765	0.978	0.734	0.758			
	C	0.737	0.803	0.910	0.662	0.769			
SPOT-1D	H	**0.931**	0.884	0.832	0.772	**0.907**	0.85	Limited to 750	2,030
	E	0.821	0.767	0.976	0.773	0.793			
	C	0.731	0.822	0.921	0.673	0.774			
MUFOLD-SSW	H	0.920	0.884	0.833	0.760	0.902	0.85	Limited to 700	150
	E	0.820	0.743	0.973	0.758	0.779			
	C	0.724	0.815	0.918	0.663	0.767			
JPred4	H	0.830	0.908	0.884	0.706	0.867	0.80	Limited to 800	110
	E	0.664	0.602	0.958	0.595	0.632			
	C	0.772	0.689	0.826	0.583	0.728			
TMPSS	H	0.907	0.888	0.842	0.752	0.897	0.84	–	**96**
	E	0.646	0.764	**0.981**	0.677	0.700			
	C	0.763	0.759	0.880	0.641	0.761			

*H, helix (DSSP classes H, G, and I); E, strand (DSSP classes E and B); C, coil (DSSP classes S, T, and blank)*.

*R, Recall; P, Precision; S, Specificity; F, F-measure. Bold fonts represent the best experimental results*.

It is worth emphasizing that this comparison shown in Table 2 is “unfair” for our experimental tool. Firstly, the existing secondary structure predictors cannot distinguish the transmembrane “H's” from non-transmembrane “H's”, whereas ours can. Secondly, some tools, such as SSpro5, uses templates, which cannot be found when making predictions about unknown structural sequences and not recommended to use under normal circumstances.

However, the tools suitable for water-soluble proteins may not be suitable for handling the residues in the transmembrane region of TMPs since they cannot distinguish transmembrane helices from non-transmembrane helices. To assess different servers' secondary structure prediction ability in the different transmembrane regions, we calculated the precision of both transmembrane and non-transmembrane residues and listed the results in Table 3. As expected, TMPSS achieved the best Q3 performance among all exemplified servers in the transmembrane region, which signified that almost all the transmembrane helices were identified by our method.

**Table 3:** Comparison of TMPSS with previous secondary structure predictors on TEST50 in the different transmembrane regions.

**Method**	**Trans SS Q3**	**Non-trans SS Q3**
SSpro5 (with templates)	0.90	**0.89**
PSIPRED 4	0.94	0.79
RaptorX-Property	0.95	0.80
Porter 5	0.95	0.81
DeepCNF	0.91	0.80
Spider3	0.95	0.80
SPOT-1D	0.95	0.81
MUFOLD-SSW	0.94	0.81
JPred4	0.90	0.75
TMPSS	**0.97**	0.78

*Trans, transmembrane region; Non-trans, non-transmembrane region. Bold fonts represent the best experimental results*.

As for topology prediction, we compared TMPSS to state-of-the-art topology predictors, including HMMTOP 2 (Tusnady and Simon, 2001), OCTOPUS (Viklund and Elofsson, 2008), TOPCONS (Tsirigos et al., 2015), Philius (Reynolds et al., 2008), PolyPhobius (Jones, 2007), SCAMPI (Bernsel et al., 2008), and SPOCTOPUS (Viklund et al., 2008). As illustrated in Table 4, TMPSS obtains the best ACC (= 0.90) and MCC (= 0.76) performance on TEST50 in the full chain among the listed methods. The most probable cause is that the joint feature learning helped two prediction tasks promote each other. According to this, the deep convolutional BiLSTM extracted the most effective information though there are only two features exploited.

**Table 4:** Comparison of TMPSS with state-of-the-art topology predictors on TEST50 in the full chain.

**Method**	**ACC**	**MCC**
HMMTOP 2	0.84	0.64
OCTOPUS	0.87	0.71
TOPCONS	0.88	0.72
Philius	0.87	0.71
PolyPhobius	0.88	0.72
SCAMPI	0.87	0.70
SPOCTOPUS	0.87	0.71
TMPSS	**0.90**	**0.76**

*Bold fonts represent the best experimental results*.

### Multi-Task Learning

Secondary structure prediction and topology structure prediction of alpha-helical TMPs are highly related tasks since the residues labeled “T” (transmembrane helix) in topology structure prediction also have the label of “H” (helix) in secondary structure prediction (Chen et al., 2002). Therefore, we put these two tasks together to support multi-task learning (Zhang and Yeung, 2012) and generated a 3-class secondary structure and a 2-class topology structure simultaneously. With the help of multi-task learning, our model's computational complexity was significantly reduced compared with other methods based on cascaded deep learning networks. The joint loss function could be formulated as follows:

(8) $$\begin{array}{r} L\left(\left\{s_{i},t_{i}\right\}\right)=\frac{\lambda_{1}}{N}\sum L_{s}\left(s_{i},{s_{i}}^{*}\right)+\frac{\lambda_{2}}{N}\sum L_{t}\left(t_{i},{t_{i}}^{*}\right)\; \end{array}$$

where $L_{s}\left(s_{i},{s_{i}}^{*}\right)=-{s_{i}}^{*}log\left(s_{i}\right)$ and $L_{t}\left(t_{i},{t_{i}}^{*}\right)=-\left[{t_{i}}^{*}log\left(t_{i}\right)+\left(1-{t_{i}}^{*}\right)log\left(1-t_{i}\right)\right]$ are respective loss functions for secondary structure and topology structure prediction, *s*_*i*_ and *t*_*i*_ are predicted probabilities (softmax output) of secondary structure labels and topology structure labels, respectively, ${s_{i}}^{*}$ and ${t_{i}}^{*}$ are ground-truth labels of secondary structure and topology structure, respectively, λ_1_ and λ_2_ are loss weight of combined loss function, and *N* is the total number of residues. Table 5 shows the effect of different loss weights (λ_1_:λ_2_) during multi-task learning on the validation dataset, and we set λ_1_ = 1, λ_2_ = 0.5 for balancing two joint feature learning tasks and regularization terms in the end.

**Table 5:** Effect of loss weight during multi-task learning.

**Loss weight (λ_1_**:**λ_2_)**	**SS Q3**	**Topo ACC**
1:0.1	0.832	0.887
1:0.3	0.833	0.892
1:0.5	**0.835**	**0.896**
1:0.7	0.825	0.892
1:1	0.830	0.894
1:5	0.811	0.889
1:10	0.794	0.892

*Bold fonts represent the best experimental results*.

### Visualization of the Features Learnt by Convolutional BiLSTM

**Figure 5:**
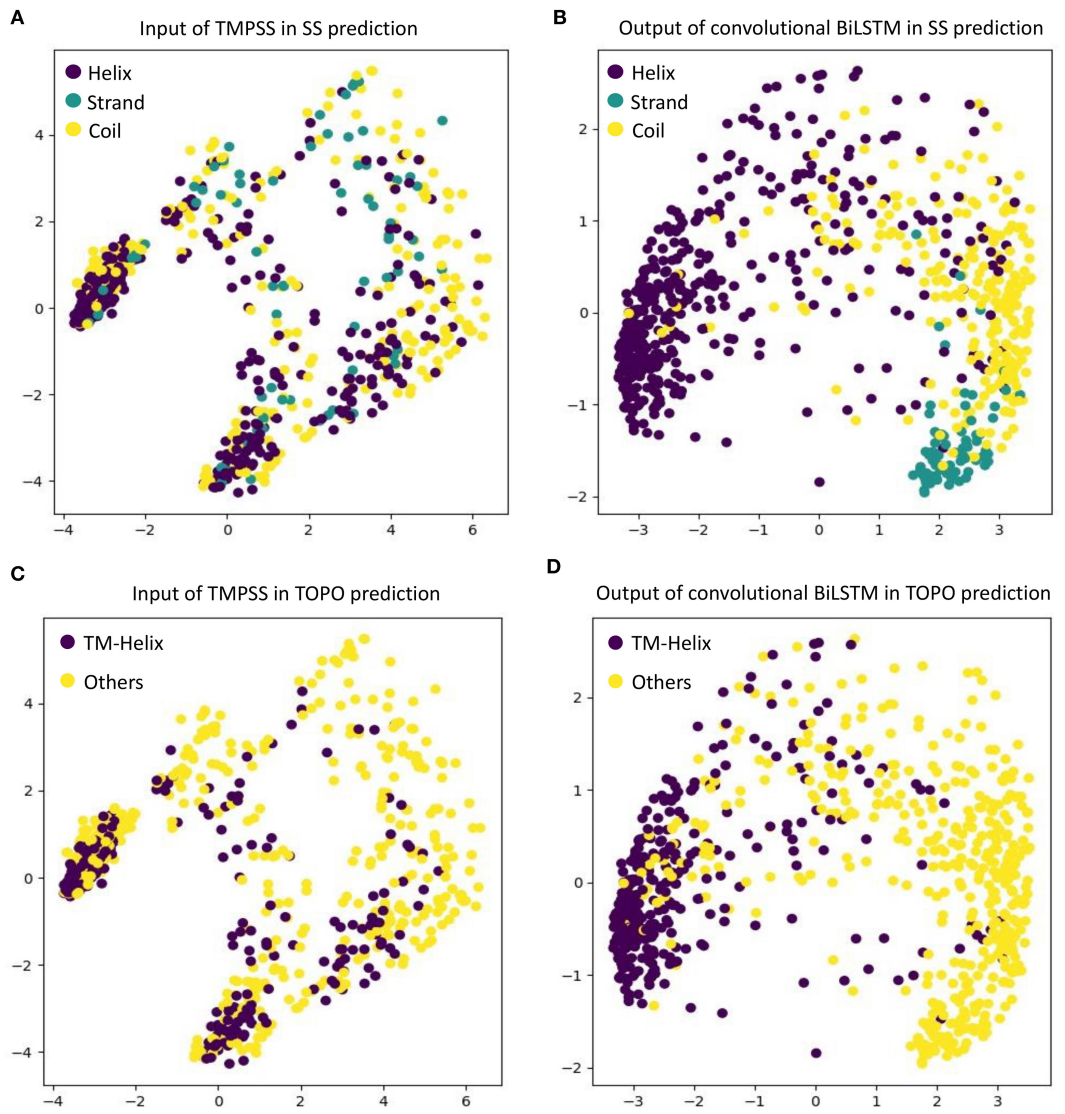
Visualize the input features and the features learned by convolutional BiLSTM, respectively, using PCA. **(A)** Input of TMPSS in SS prediction. **(B)** Output of convolutional BiLSTM in SS prediction. **(C)** Input of TMPSS in TOPO prediction. **(D)** Output of convolutional BiLSTM in TOPO prediction.

As an automatic feature extraction process, deep learning can learn high-level abstract features from original inputs (Farias et al., 2016). To further explore the effectiveness of convolutional BiLSTM, Principal Component Analysis (PCA) (Shlens, 2014) was utilized to visualize the input features and each LSTM unit's output in the last bidirectional layer with TEST50. Figure 5 shows the PCA scatter diagrams before and after TEST50 was fed into our network, respectively.

As described earlier, the input data had 52 features (i.e., 52 dimensions). PCA reduced the input features' dimensionality to two principal dimensions and visualized it. As we can see in Figures 5A,C, no clear cluster can be found. However, after feeding the data into the convolutional BiLSTM that contains 1,400 dimensions (twice of the unit number in a simple LSTM) at the top layer, the data points showed apparent clustering tendency (see Figures 5B,D). This visualization experiment strongly proved the feature extraction efficiency of the convolutional BiLSTM.

It is worth mentioning that since multi-task joint feature learning was performed in our network, the label-based visualization results also revealed the internal relation between secondary structure prediction and topology structure prediction. We found that the points representing “helices” of secondary structure and the ones representing “transmembrane helices” of topology structure have almost completely overlapping distributions under different label-orientated predictions. This experimental phenomenon also directly confirmed the strong correlation between the two prediction tasks and the necessity and effectiveness of multi-task learning.

More results, such as the prediction performance analysis at the residue level, feature analysis, implementation details of multi-task learning, implementation details of attention mechanism, and an ablation study, can be found in the Supplementary Material.

### Attention Mechanism

The attention mechanism can stimulate the model extracting features more effectively, speeding up reaching or even improving the best performance of prediction (Choi et al., 2016). To verify the effect of various binding ways of attention mechanism, which acted as a simple full-connect layer in our model, we combined it with different network layers, and the results are shown in Table 6. It can be seen that when we attached an attention layer to BiLSTM layers, the prediction results (SS Q3 = 0.835 and Topo ACC = 0.896) were better than doing the same thing to multiscale CNNs or the Dropout layer as expected. One reason could be that the attention mechanism enhanced the process of feature extraction. Another reason could be that BiLSTM layers just learned the most abundant contextual features, making it achieve the best effect when combining attention layer with BiLSTM layers.

**Table 6:** Effect of different combination ways of the attention mechanism on TEST50.

**Model**	**SS Q3**	**Topo ACC**
Attention with multiscale CNNs	0.826	0.893
Attention with BiLSTM	**0.835**	**0.896**
Attention with dropout	0.742	0.866

*Bold fonts represent the best experimental results*.

### Ablation Study

To discover whether a certain component of our proposed method was vital or necessary, we carried out an ablation study by removing some network elements in this section. The experiments performed in our ablation study shared the same features and hyperparameters. From the results on TEST50 presented in Table 7, we found that those BiLSTM layers were the most contributing and effective component in our model since the Q3 accuracy of secondary structure prediction dropped to 75.9% when we roughly removed this part from the network. Multiscale CNNs were also essential for good performance as they were particularly good at dealing with local information of protein sequences. Furthermore, multi-task learning and attention mechanism were necessary at the same time because their application made contributions to the robustness of our method with the proof of study results.

**Table 7:** An ablation study on TEST50.

**Model**	**SS Q3**	**Topo ACC**
Without multiscale CNNs	0.832	0.895
Without BiLSTM layers	0.759	0.743
Without multi-task learning	0.825	0.891
Without attention mechanism	0.828	0.892
TMPSS	**0.835**	**0.896**

*Bold fonts represent the best experimental results*.

### Case Study

**Figure 6:**
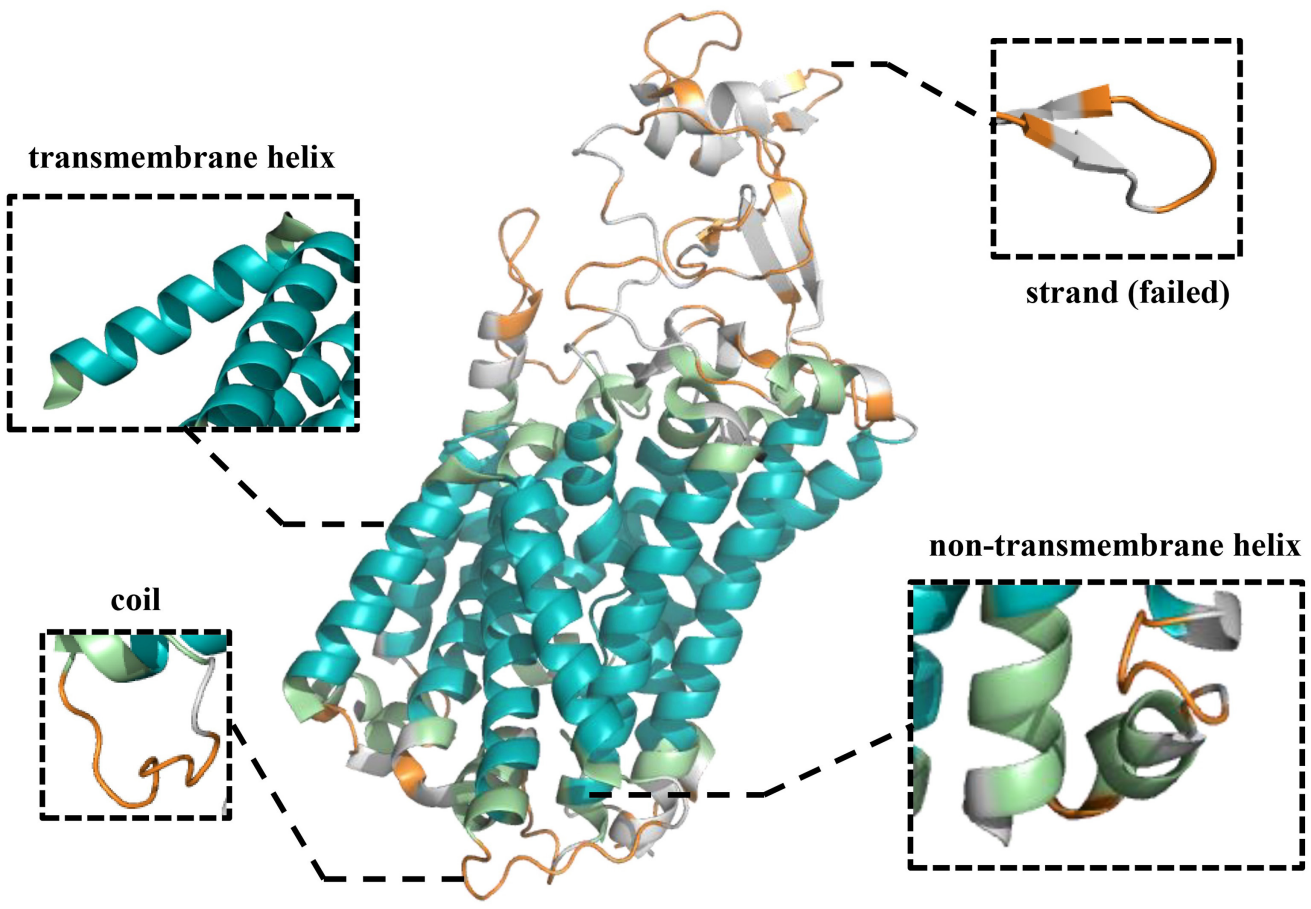
Visualization of secondary structure and topology structure prediction results generated by TMPSS with PyMOL: take 6KKT_A as an example.

To further demonstrate the effectiveness of TMPSS on predicting the secondary structure and topology structure of alpha-helical TMPs, we randomly took 6KKT_A as an example of our case study. 6KKT is a kind of transport protein of *Homo sapiens* released on 2019-10-23 that plays vital roles in cell volume regulation, ion transport, and salt reabsorption in the kidney (Liu et al., 2019). The prediction result of TMPSS is visualized in Figure 6 using PyMOL (DeLano, 2002).

As can be seen, our model correctly identified the helices in the transmembrane region (colored blue) and the non-transmembrane region (colored green). Additionally, most of the coils in the non-transmembrane region (colored orange) were also successfully distinguished.

## Conclusion

In this study, we proposed a deep learning-based predictor, TMPSS, to predict the secondary structure and topology structure of alpha-helical TMPs from primary sequences. TMPSS's Q3 accuracy of secondary structure prediction in the full chain performed on par with the state-of-the-art methods statistically, and our model had the highest output efficiency with no length restriction of input at the same time. Moreover, our method achieved the best Q3 performance in the transmembrane region and significantly outperformed other topology structure predictors on the independent dataset TEST50.

TMPSS applied a deep learning network with grouped multiscale CNNs and stacked attention-enhanced BiLSTM layers for capturing local and global contexts. Multi-task learning was exploited to improve prediction performance and reduce our method's computational expense by considering the interactions between different protein properties. A series of visualization experiments and comparative tests was taken to verify the validity of the model components mentioned above.

Furthermore, we implemented TMPSS as a publicly available predictor for the research community. The pre-trained model and the datasets we used in this paper could be downloaded at https://github.com/NENUBioCompute/TMP-SS. Finally, we sincerely hope that the predictor and the support materials we released in this study will help the researchers who need them.

## Data Availability Statement

The original contributions presented in the study are included in the article/Supplementary Materials, further inquiries can be directed to the corresponding authors.

## Author Contributions

ZL, YGo, and YB conceived the idea of this research, collected the data, implemented the predictor, and wrote the manuscript. ZL and YGu tuned the model and tested the predictor. HW and GL supervised the research and reviewed the manuscript. All authors contributed to the article and approved the submitted version.

## Conflict of Interest

The authors declare that the research was conducted in the absence of any commercial or financial relationships that could be construed as a potential conflict of interest. The reviewer P-FD declared a shared affiliation, with no collaboration, with the author YGo to the handling editor at the time of the review.
