# Supplementary material for: TMPSS: A Deep Learning-Based Predictor for Secondary Structure and Topology Structure Prediction of Alpha-Helical Transmembrane Proteins
Source: Front Bioeng Biotechnol. 2021 Jan 25;8:629937. doi: 10.3389/fbioe.2020.629937 (PMC7869861; doi:10.3389/fbioe.2020.629937)
Supplement: Supplementary file 1 [file Data_Sheet_1.zip › Supplementary Materials.docx]

Supplementary Material

**1Introduction**

Progress in the structure prediction for membrane proteins is slower than soluble proteins (Xiao and Shen, 2015). At present, state-of-the-art methods aiming at predicting secondary structure based on primary sequences listed in **Supplementary Table 1**, are all trained on soluble proteins-specific datasets. However, none of those mentioned methods can simultaneously predict the secondary structure and topology structure of alpha-helical TMPs. More specifically, existing tools could not distinguish transmembrane helices of TMPs from non-transmembrane ones and, in-term, would weakenthe TMPs’ structure prediction specificity. Another common challenge among the available methods is that features fed into these models are often too miscellaneous, making the model prediction low efficient and even difficultfor users to understand. Thus, a more suitable and practical tool for assisting the structure prediction of TMPs is greatly needed.

| **Method** | **Year** | **Outputs** | **Algorithm** |  |  |
| --- | --- | --- | --- | --- | --- |
| SPINE X | 2012 | protein secondary structure (Q3), backbone torsion angles, and solvent accessibility | ANN |  |  |
| SSpro/ACCpro 5 | 2014 | protein secondary structure (Q3, Q8) and relative solvent accessibility | BiRNN |  |  |
| JPred4 | 2015 | protein secondary structure (Q3), solvent accessibility, and coiled-coil regions | HMM |  |  |
| DeepCNF | 2016 | protein secondary structure (Q3, Q8) | DeepCNF |  |  |
| DCRNN | 2016 | protein secondary structure (Q8) | CNN, BiGRU |  |  |
| RaptorX-Property | 2016 | secondary structure (Q3, Q8), solvent accessibility, and disorder regions | DeepCNF |  |  |
| Spider3 | 2017 | protein secondary structure (Q3), backbone angles, contact numbers, and solvent accessibility | LSTM, BiRNN |  |  |
| CNNH_PSS | 2018 | protein secondary structure (Q8) | CNN |  |  |
| DeepACLSTM | 2019 | protein secondary structure (Q8) | CNN, BiLSTM |  |  |
| DeepPrime2Sec | 2019 | protein secondary structure (Q8) | CNN, BiLSTM |  |  |
| Porter 5 | 2019 | protein secondary structure (Q3, Q8) | CNN, BiLSTM |  |  |
| Hermes | 2019 | protein secondary structure (Q3) | MLP, RNN, CNN, BiLSTM |  |  |
| PSIPRED 4 | 2019 | protein secondary structure (Q3) | ANN |  |  |
| SPOT-1D | 2019 | protein secondary structure (Q3, Q8), backbone angles, half-sphere exposure, contact numbers, and relative solvent accessibility | ResNets, LSTM-BiRNN |  |  |
| MUFOLD-SSW | 2020 | protein secondary structures (Q3, Q8), torsion angles, beta-turns and gamma-turns | Deep3I |  |  |

**SupplementaryTable 1.** Comparison among existing methods for predicting secondary structure based on primary sequences.

**2 Materials and Methods**

**2.1 Features and input Encoding**

We adopted a 21-dimensional matrix $O_{onehot}$as our second set contained a simple one-hot encoding of 20 positions with one $NoSeq$label (see **Supplementary Figure1**). The past research suggested that one-hot encoding was straightforward to generate and has been successfully used in protein structure prediction associated tasks (He et al., 2018). This coding used 19 dimensional ‘0’ vector with a ‘1’ representing AA at the index of a particular protein sequence. We mapped each protein fragment sliced by the sliding window with this encoding strategy into an undisturbed coding within local position information.


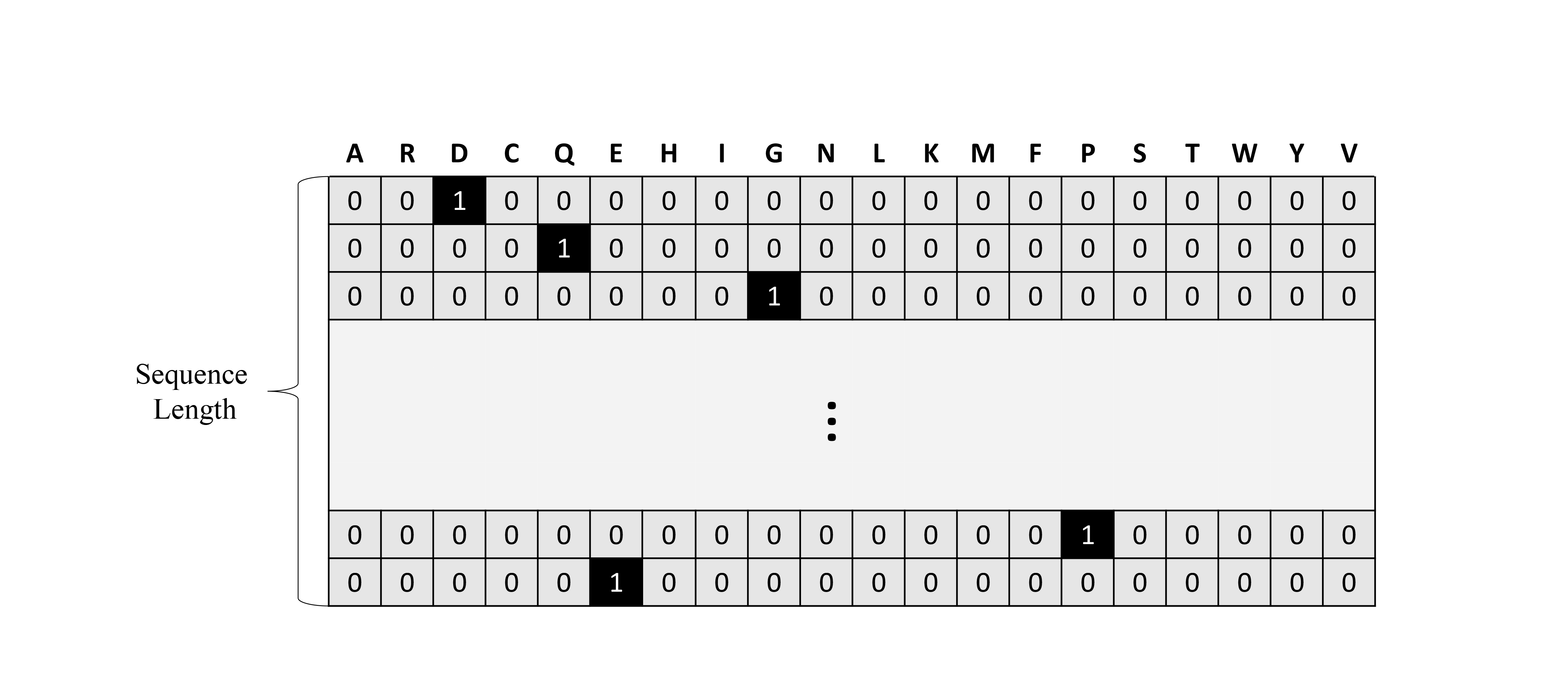


**Supplementary Figure1.**One-hot code of protein residues.

**2.3 Model Design**

**2.3.1 Grouped Multiscale CNNs**

As shown in **Figure 2**, a grouped multiscale convolutional layer was implemented as the second component of our network. Two independent CNN layers and multiscale kernels were used while extracting the sequence feature and the profile feature, respectively, so that modeling local featureswould not be affected by different feature magnitude(Yip and Hu, 2018). The given sliding window-sliced sequence with one-hot encoding and HHblits profile features can be expressed as the following formula:

$$\begin{aligned} \boldsymbol{X}\boldsymbol{=}\left[ \boldsymbol{x}_{\boldsymbol{1}}\boldsymbol{,}\boldsymbol{x}_{\boldsymbol{2}}\boldsymbol{, \ldots,}\boldsymbol{x}_{\boldsymbol{i}}\boldsymbol{, \ldots,}\boldsymbol{x}_{\boldsymbol{L}} \right]\#\left( \mathrm{AUTONUM} \right) \end{aligned}$$

where $x_{i}$($\in\mathbb{R}^{52}$) is the preprocessed 52-dimensional input vector of the $i$-th AA with the sliding window length of $L$residues. In our experiments, the Rectified Linear Unit (ReLU) (Dahl et al., 2013) was utilized as an activation function to enhance the nonlinear relationship between neural network layers.

$$\begin{aligned} \boldsymbol{c}_{\boldsymbol{i}}\boldsymbol{=K*}\boldsymbol{x}_{\boldsymbol{i:i+k-1}}\boldsymbol{=ReLU}\left( \boldsymbol{w*}\boldsymbol{x}_{\boldsymbol{i:i+k-1}}\boldsymbol{+b} \right)\#\left( \mathrm{AUTONUM} \right) \end{aligned}$$

Formula$\left( 2 \right)$ shows the internal calculation of a certain CNN layer where $K$($\in\mathbb{R}^{k*52}$) represents a convolutional kernel, $k$represents the size of kernel along with the protein sequence, $w$represents the weight matrix and $b$represents the bias term of a single unit. Each kernel $K$goes through the whole input vector $x_{i}$and generates a corresponding output each time. In this paper, we used two CNN layers with $k$= 3 and 5 for one-hot encoding features and three CNN layers with $k$=3, 5, and 7 for HHblits profile features. The outputs of these CNN layers were merged as the local contexts of input features.

**2.3.2 Attention-enhanced BiLSTM**

Apart from local information, long-term dependencies also take a non-negligible proportion in protein sequences. As a variant of recurrent neural networks (RNN), Bidirectional Long Short Term Memory (BiLSTM) can model long sequences and learn a reasonable vector representation. Therefore, we implemented BiLSTM layers at the end of CNN layers to learn and store long-range information. The internal structure of a single LSTM unit is shown in **Supplementary Figure2.**

Formula$\left( 3 \right)$ to Formula$\left( 10 \right)$ represent the forward recursions for a single LSTM layer, where ⊙stands forthe matrix multiplication, $x_{t}$equals the input from the previous layer, $h_{t-rec}$means the output forwarded to the next time slice, and $h_{t}$ is the passed upwards in multilayer LSTMs (Sønderby and Winther, 2014).

$$\begin{aligned} \boldsymbol{i}_{\boldsymbol{t}}\boldsymbol{=\sigma}\left( \boldsymbol{x}_{\boldsymbol{t}}\boldsymbol{W}_{\boldsymbol{x}_{\boldsymbol{i}}}\boldsymbol{+}\boldsymbol{h}_{\boldsymbol{t-1}}\boldsymbol{W}_{\boldsymbol{h}_{\boldsymbol{i}}}\boldsymbol{+}\boldsymbol{b}_{\boldsymbol{i}} \right)\boldsymbol{\#}\left( AUTONUM \backslash* Arabic \right) \end{aligned}$$

$$\begin{aligned} \boldsymbol{f}_{\boldsymbol{t}}\boldsymbol{=\sigma}\left( \boldsymbol{x}_{\boldsymbol{t}}\boldsymbol{W}_{\boldsymbol{xf}}\boldsymbol{+}\boldsymbol{h}_{\boldsymbol{t-1}}\boldsymbol{W}_{\boldsymbol{hf}}\boldsymbol{+}\boldsymbol{b}_{\boldsymbol{f}} \right)\boldsymbol{\#}\left( AUTONUM \backslash* Arabic \right) \end{aligned}$$

$$\begin{aligned} \boldsymbol{o}_{\boldsymbol{t}}\boldsymbol{=\sigma}\left( \boldsymbol{x}_{\boldsymbol{t}}\boldsymbol{W}\boldsymbol{x}_{\boldsymbol{o}}\boldsymbol{+}\boldsymbol{h}_{\boldsymbol{t-1}}\boldsymbol{W}_{\boldsymbol{h}_{\boldsymbol{0}}}\boldsymbol{+}\boldsymbol{b}_{\boldsymbol{o}} \right)\boldsymbol{\#}\left( AUTONUM \backslash* Arabic \right) \end{aligned}$$

$$\begin{aligned} \boldsymbol{g}_{\boldsymbol{t}}\boldsymbol{=}\tanh\left( \boldsymbol{x}_{\boldsymbol{t}}\boldsymbol{W}_{\boldsymbol{xg}}\boldsymbol{+}\boldsymbol{h}_{\boldsymbol{t-1}}\boldsymbol{W}_{\boldsymbol{hg}}\boldsymbol{+}\boldsymbol{b}_{\boldsymbol{g}} \right)\boldsymbol{\#}\left( AUTONUM \backslash* Arabic \right) \end{aligned}$$

$$\begin{aligned} \boldsymbol{c}_{\boldsymbol{t}}\boldsymbol{=}\boldsymbol{f}_{\boldsymbol{t}}\boldsymbol{\odot}\boldsymbol{c}_{\boldsymbol{t-1}}\boldsymbol{+}\boldsymbol{i}_{\boldsymbol{t}}\boldsymbol{\odot}\boldsymbol{g}_{\boldsymbol{t}}\boldsymbol{\#}\left( AUTONUM \backslash* Arabic \right) \end{aligned}$$

$$\begin{aligned} \boldsymbol{h}_{\boldsymbol{t}}\boldsymbol{=}\boldsymbol{o}_{\boldsymbol{t}}\boldsymbol{\odot}\tanh\left( \boldsymbol{c}_{\boldsymbol{t}} \right)\boldsymbol{\#}\left( \mathrm{AUTONUM} \right) \end{aligned}$$

$$\begin{aligned} \boldsymbol{h}_{\boldsymbol{t}\boldsymbol{-}\boldsymbol{rec}}\boldsymbol{=}\boldsymbol{h}_{\boldsymbol{t}}\boldsymbol{+feedforwardnet}\left( \boldsymbol{h}_{\boldsymbol{t}} \right)\boldsymbol{\#}\left( \mathrm{AUTONUM} \right) \end{aligned}$$

$$\begin{aligned} \boldsymbol{\sigma}\left( \boldsymbol{z} \right)\boldsymbol{=}\frac{\boldsymbol{1}}{\boldsymbol{1+}\exp\left( \boldsymbol{-z} \right)}\boldsymbol{\#}\left( \mathrm{AUTONUM} \right) \end{aligned}$$

Unfortunately, the number of residues in a single sequence can easily exceed 1000, and it is difficult and challenging for BiLSTM to extract long-term dependencies from such extremely long input sequences. Therefore, we tried to optimize our method with an attention mechanism (Firat et al., 2016) to help BiLSTM focus on the region, which is more important and deserves relatively more attention (Choi et al., 2016). As shown in Formula$\left( 11 \right)$, the implementation of the attention mechanism enables the network to re-assign the weight matrix $W_{att}$of the input vector $V$and provide a new vector $V^{'}$. It endowsthe model with two abilities: automatically focusing on the units that have a decisive effecton classification andcapturingthe most important semantic information in a sequence (Zhou et al., 2016).

$$\begin{aligned} \boldsymbol{V}^{\boldsymbol{'}}\boldsymbol{=}\boldsymbol{W}_{\boldsymbol{att}}\boldsymbol{\odot V\#}\left( \mathrm{AUTONUM} \right) \end{aligned}$$

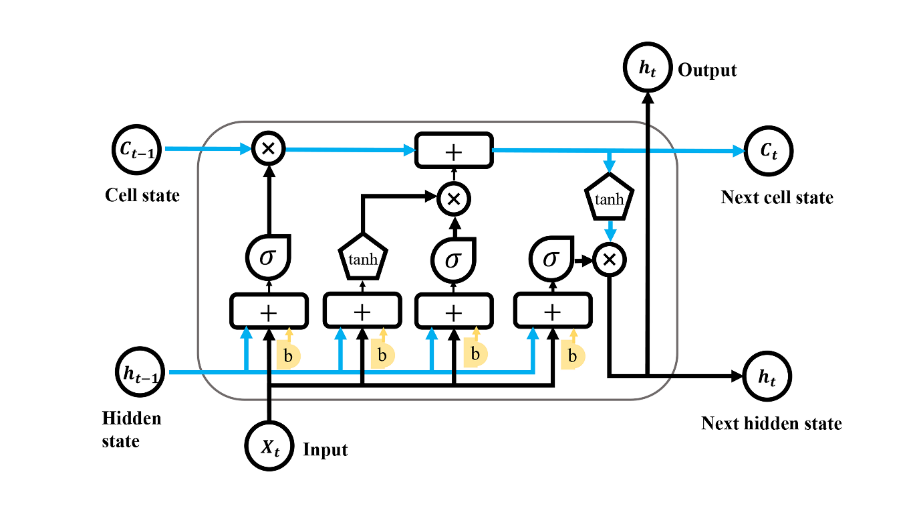


**Supplementary Figure2.**The internal structure of a single LSTM unit.

**2.3.3 Implementation Details**

Our model was implemented, trained, and tested using the open-source software library Keras and Tensorflowon an Nvidia 1080Ti GPU. Main hyperparameters such as sliding window length, training dropout rate, number of LSTM units were explored, and an early stopping strategy and a save-best strategy were adopted. When the validation loss did not reduce in ten epochs during training time, the training process would stop, and the best model parameters would be saved. In all cases, the weights were initialized by default setting in Keras; the parameters were trained using Adam optimizer to change the learning rate during model training dynamically. Furthermore, batch normalization layers and a Dropout layer (rate = 0.30) were utilized since they were both skilled in avoiding networks from overfitting and improving the training process's speed. In this study, we set 19 residues as the sliding window's length and put 700 units in each LSTM layer according to the hyperparameter tuning results.

All the experiments were performed on an Nvidia 1080Ti GPU, and with the help of Tensorboard, we visualized dynamic loss and metric curves of trainingand validation set. As shown in **Supplementary Figure3**, the curves of two different tasks (secondary structure prediction and topology structure prediction) converged in the correct direction thanks to the learning strategies mentioned above.


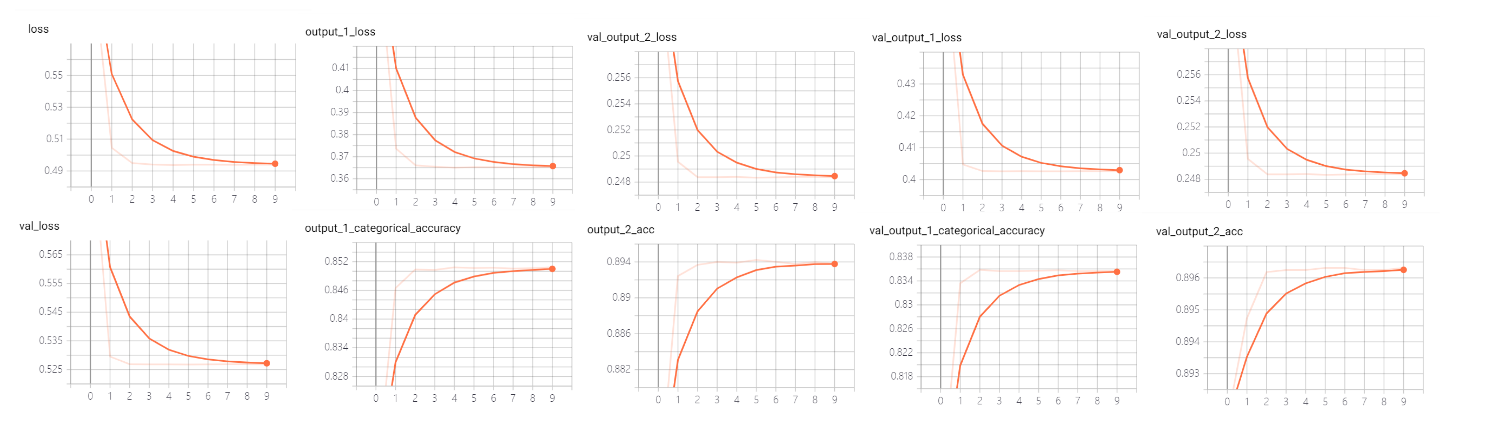


**Supplementary Figure3.**Loss and metric curves of training and validation set generated by Tensorboard.The curves of two different tasks (secondary structure prediction and topology structure prediction) converged in the correct direction based on the learning strategies mentioned above.

**3 Results**

**3.1 Feature Analysis**

Input features determine the upper-performancelimit of deep learning methods (Tishby and Zaslavsky, 2015), but too many features may increase the network's complexity and computational cost. To investigate various features’ contribution to TMPSS, we tested all independent features used in our model and their combination on the validation dataset.

**Supplementary Table 2** demonstrates that all two features (one-hot encoding and HHblits profile) contained informative contexts for secondary structure (SS) and topology structure (Topo) prediction of alpha-helical TMPs, and between them HHblits profile achieved the best overall results (SS Q3 = 0.820 and Topo ACC = 0.889). The experimental investigation also indicated that our method reached the most considerable performance (SS Q3 = 0.835 and Topo ACC = 0.896) when these two features were combined.

| **Feature** | **SS Q3** | **Topo ACC** |
| --- | --- | --- |
| One-hot | 0.742 | 0.866 |
| HHblits | 0.820 | 0.889 |
| One-hot + HHblits | **0.835** | **0.896** |

**Supplementary Table 2.**Prediction performance based on individual input features and their various combinations.

**References**

Asgari, E., Poerner, N., McHardy, A., and Mofrad, M. (2019). DeepPrime2Sec: Deep Learning for Protein Secondary Structure Prediction from the Primary Sequences. *bioRxiv***,** 705426.

Bliss, L., Pascoe, B., and Sheppard, S.K. (2019). Hermes: an ensemble machine learning architecture for protein secondary structure prediction. *bioRxiv***,** 640656.

Buchan, D.W., and Jones, D.T. (2019). The PSIPRED protein analysis workbench: 20 years on. *Nucleic acids research* 47(W1)**,** W402-W407.

Chen, C.P., Kernytsky, A., and Rost, B. (2002). Transmembrane helix predictions revisited. *Protein Science* 11(12)**,** 2774-2791.

Choi, E., Bahadori, M.T., Sun, J., Kulas, J., Schuetz, A., and Stewart, W. (Year). "Retain: An interpretable predictive model for healthcare using reverse time attention mechanism", in: *Advances in Neural Information Processing Systems*), 3504-3512.

Dahl, G.E., Sainath, T.N., and Hinton, G.E. (Year). "Improving deep neural networks for LVCSR using rectified linear units and dropout", in: *2013 IEEE international conference on acoustics, speech and signal processing*: IEEE), 8609-8613.

Drozdetskiy, A., Cole, C., Procter, J., and Barton, G.J. (2015). JPred4: a protein secondary structure prediction server. *Nucleic acids research* 43(W1)**,** W389-W394.

Fang, C., Li, Z., Xu, D., and Shang, Y. (2020). MUFold-SSW: a new web server for predicting protein secondary structures, torsion angles and turns. *Bioinformatics* 36(4)**,** 1293-1295.

Faraggi, E., Zhang, T., Yang, Y., Kurgan, L., and Zhou, Y. (2012). SPINE X: improving protein secondary structure prediction by multistep learning coupled with prediction of solvent accessible surface area and backbone torsion angles. *Journal of computational chemistry* 33(3)**,** 259-267.

Firat, O., Cho, K., and Bengio, Y. (2016). Multi-way, multilingual neural machine translation with a shared attention mechanism. *arXiv preprint arXiv:1601.01073*.

Fu, L., Niu, B., Zhu, Z., Wu, S., and Li, W. (2012). CD-HIT: accelerated for clustering the next-generation sequencing data. *Bioinformatics* 28(23)**,** 3150-3152.

Guo, Y., Li, W., Wang, B., Liu, H., and Zhou, D. (2019). DeepACLSTM: deep asymmetric convolutional long short-term memory neural models for protein secondary structure prediction. *BMC bioinformatics* 20(1)**,** 341.

Hanson, J., Paliwal, K., Litfin, T., Yang, Y., and Zhou, Y. (2019). Improving prediction of protein secondary structure, backbone angles, solvent accessibility and contact numbers by using predicted contact maps and an ensemble of recurrent and residual convolutional neural networks. *Bioinformatics* 35(14)**,** 2403-2410.

He, F., Wang, R., Li, J., Bao, L., Xu, D., and Zhao, X. (2018). Large-scale prediction of protein ubiquitination sites using a multimodal deep architecture. *BMC systems biology* 12(6)**,** 109.

Heffernan, R., Yang, Y., Paliwal, K., and Zhou, Y. (2017). Capturing non-local interactions by long short-term memory bidirectional recurrent neural networks for improving prediction of protein secondary structure, backbone angles, contact numbers and solvent accessibility. *Bioinformatics* 33(18)**,** 2842-2849.

Kabsch, W., and Sander, C. (1983). DSSP: definition of secondary structure of proteins given a set of 3D coordinates. *Biopolymers* 22**,** 2577-2637.

Li, Z., and Yu, Y. (2016). Protein secondary structure prediction using cascaded convolutional and recurrent neural networks. *arXiv preprint arXiv:1604.07176*.

Magnan, C.N., and Baldi, P. (2014). SSpro/ACCpro 5: almost perfect prediction of protein secondary structure and relative solvent accessibility using profiles, machine learning and structural similarity. *Bioinformatics* 30(18)**,** 2592-2597.

Sønderby, S.K., and Winther, O. (2014). Protein secondary structure prediction with long short term memory networks. *arXiv preprint arXiv:1412.7828*.

Tishby, N., and Zaslavsky, N. (Year). "Deep learning and the information bottleneck principle", in: *2015 IEEE Information Theory Workshop (ITW)*: IEEE), 1-5.

Torrisi, M., Kaleel, M., and Pollastri, G. (2019). Deeper Profiles and Cascaded Recurrent and Convolutional Neural Networks for state-of-the-art Protein Secondary Structure Prediction. *Scientific reports* 9(1)**,** 1-12.

Wang, S., Li, W., Liu, S., and Xu, J. (2016a). RaptorX-Property: a web server for protein structure property prediction. *Nucleic acids research* 44(W1)**,** W430-W435.

Wang, S., Peng, J., Ma, J., and Xu, J. (2016b). Protein secondary structure prediction using deep convolutional neural fields. *Scientific reports* 6(1)**,** 1-11.

Xiao, F., and Shen, H.-B. (2015). Prediction enhancement of residue real-value relative accessible surface area in transmembrane helical proteins by solving the output preference problem of machine learning-based predictors. *Journal of chemical information and modeling* 55(11)**,** 2464-2474.

Yip, C., and Hu, H. (Year). "Grouped multi-task CNN for facial attribute recognition", in: *2018 24th International Conference on Pattern Recognition (ICPR)*: IEEE), 272-277.

Zhang, Y., and Yeung, D.-Y. (2012). A convex formulation for learning task relationships in multi-task learning. *arXiv preprint arXiv:1203.3536*.

Zhou, J., Wang, H., Zhao, Z., Xu, R., and Lu, Q. (2018). CNNH_PSS: protein 8-class secondary structure prediction by convolutional neural network with highway. *BMC bioinformatics* 19(4)**,** 60.

Zhou, P., Shi, W., Tian, J., Qi, Z., Li, B., Hao, H., et al. (Year). "Attention-based bidirectional long short-term memory networks for relation classification", in: *Proceedings of the 54th annual meeting of the association for computational linguistics (volume 2: Short papers)*), 207-212.
