# Supplementary figures and images for: TMPSS: A Deep Learning-Based Predictor for Secondary Structure and Topology Structure Prediction of Alpha-Helical Transmembrane Proteins
Source: Front Bioeng Biotechnol. 2021 Jan 25;8:629937. doi: 10.3389/fbioe.2020.629937 (PMC7869861; doi:10.3389/fbioe.2020.629937)

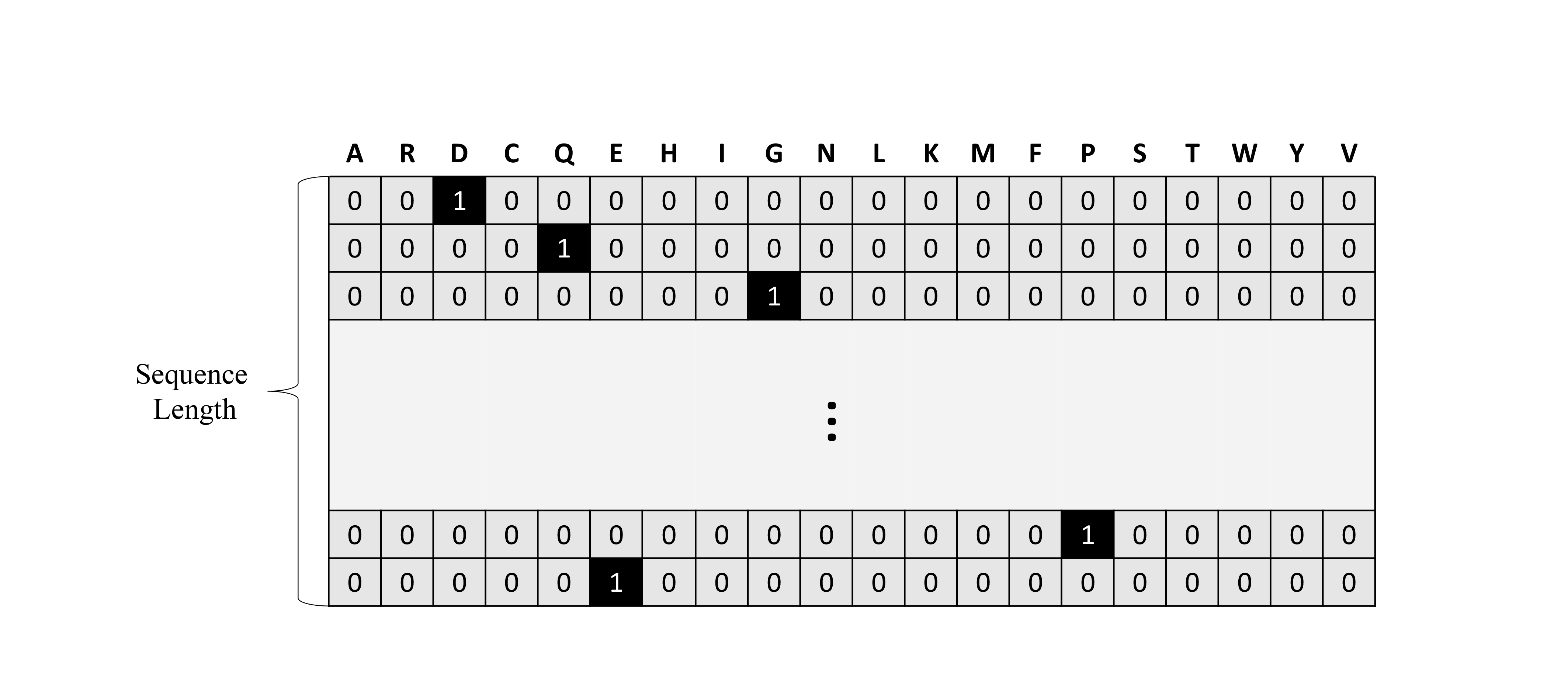

Supplement: Supplementary file 1 [file Data_Sheet_1.zip › images/Supplementary Figure 1.jpg]

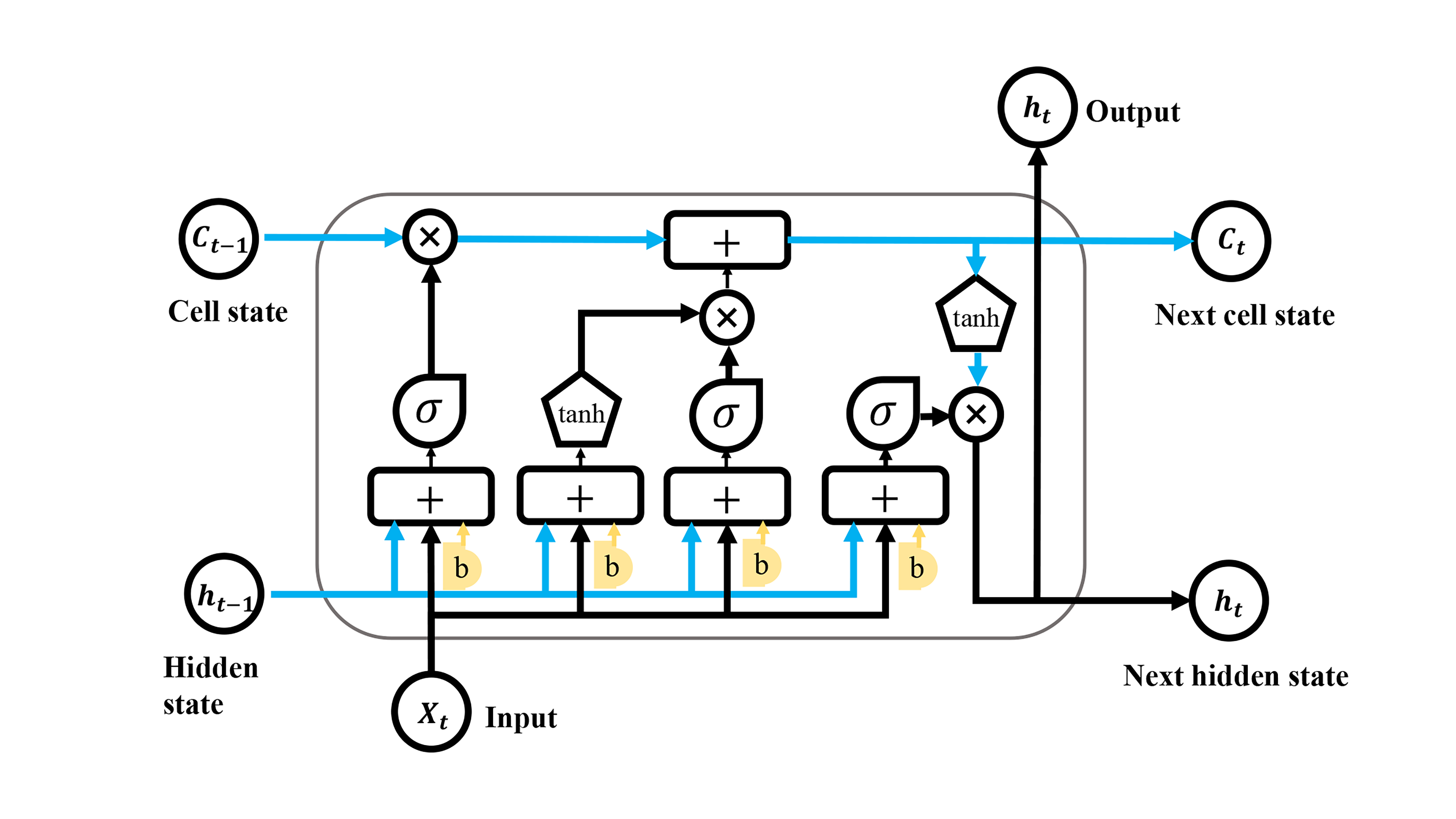

Supplement: Supplementary file 1 [file Data_Sheet_1.zip › images/Supplementary Figure 2.jpg]

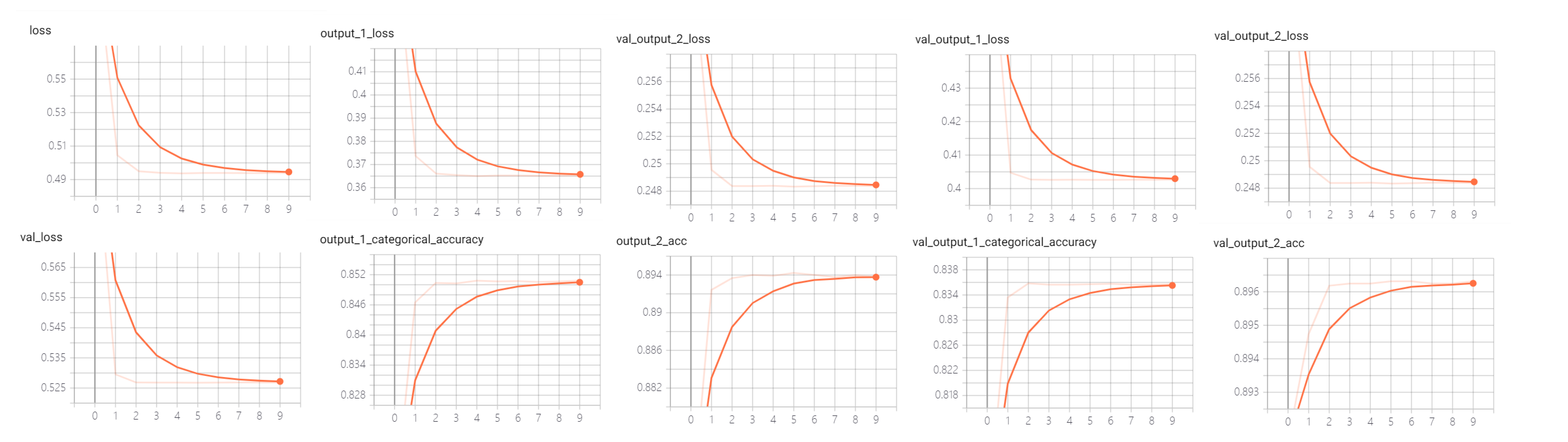

Supplement: Supplementary file 1 [file Data_Sheet_1.zip › images/Supplementary Figure 3.jpg]
